# Phase I vaccination trial of SYT-SSX junction peptide in patients with disseminated synovial sarcoma

**DOI:** 10.1186/1479-5876-3-1

**Published:** 2005-01-12

**Authors:** Satoshi Kawaguchi, Takuro Wada, Kazunori Ida, Yuriko Sato, Satoshi Nagoya, Tomohide Tsukahara, Sigeharu Kimura, Hiroeki Sahara, Hideyuki Ikeda, Kumiko Shimozawa, Hiroko Asanuma, Toshihiko Torigoe, Hiroaki Hiraga, Takeshi Ishii, Shin-ichiro Tatezaki, Noriyuki Sato, Toshihiko Yamashita

**Affiliations:** 1 Department of Orthopaedic Surgery, Sapporo Medical University School of Medicine, Sapporo, Japan; 2 Department of Pathology, Sapporo Medical University School of Medicine, Sapporo, Japan; 3 Marine Biomedical Institute, Sapporo Medical University School of Medicine, Rishirifuji, Japan; 4 Cancer Vaccine Laboratory, Innovation Plaza Hokkaido, Japan Science and Technology Corporation, Sapporo, Japan; 5 Division of Orthopedics, National Hospital Organization Hokkaido Cancer Center, Sapporo, Japan; 6 Division of Orthopaedic Surgery, Chiba Cancer Center Hospital, Chiba, Japan

**Keywords:** Synovial sarcoma, SYT-SSX, antigenic peptide, vaccination, Phase I trial

## Abstract

**Background:**

Synovial sarcoma is a high-grade malignant tumor of soft tissue, characterized by the specific chromosomal translocation t(X;18), and its resultant SYT-SSX fusion gene. Despite intensive multimodality therapy, the majority of metastatic or relapsed diseases still remain incurable, thus suggesting a need for new therapeutic options. We previously demonstrated the antigenicity of SYT-SSX gene-derived peptides by in vitro analyses. The present study was designed to evaluate in vivo immunological property of a SYT-SSX junction peptide in selected patients with synovial sarcoma.

**Methods:**

A 9-mer peptide (SYT-SSX B: GYDQIMPKK) spanning the SYT-SSX fusion region was synthesized. Eligible patients were those (i) who have histologically and genetically confirmed, unresectable synovial sarcoma (SYT-SSX1 or SYT-SSX2 positive), (ii) HLA-A*2402 positive, (iii) between 20 and 70 years old, (iv) ECOG performance status between 0 and 3, and (v) who gave informed consent. Vaccinations with SYT-SSX B peptide (0.1 mg or 1.0 mg) were given subcutaneously six times at 14-day intervals. These patients were evaluated for DTH skin test, adverse events, tumor size, tetramer staining, and peptide-specific CTL induction.

**Results:**

A total of 16 vaccinations were carried out in six patients. The results were (i) no serious adverse effects or DTH reactions, (ii) suppression of tumor progression in one patient, (iii) increases in the frequency of peptide-specific CTLs in three patients and a decrease in one patient, and (iv) successful induction of peptide-specific CTLs from four patients.

**Conclusions:**

Our findings indicate the safety of the SYT-SSX junction peptide in the use of vaccination and also give support to the property of the peptide to evoke in vivo immunological responses. Modification of both the peptide itself and the related protocol is required to further improve the therapeutic efficacy.

## Background

Synovial sarcoma is a relatively rare, high-grade malignant tumor of soft tissue, characterized by biphasic or monophasic histology, specific chromosomal translocation t(X;18), and its resultant SYT-SSX fusion gene[1]. This tumor affects mostly adolescents and young adults. The 5-year survival rates of patients with this localized disease have ranged from 66% to 80% in the current literature [2-5]. However, the majority of metastatic or relapsed diseases still remain incurable despite intensive multimodality therapy. Therefore there is a need for additional new therapeutic options other than conventional surgery, radiotherapy, and chemotherapy.

Vaccination of tumor antigenic peptide serves as a commonly accepted method in anti-cancer immunotherapy[6,7]. This is based on the rationale that T cells recognize antigenic peptide in the context of MHC molecules on the tumor cell or antigen presenting cells through the T cell receptor, which elicits subsequent anti-tumor immune responses. Identification of antigenic peptides recognized T cells enabled us to apply vaccination trials to a variety of tumors, except bone and soft tissue sarcomas[8].

Currently tumor specific chromosomal translocations are defined in leukemias, lymphomas, and sarcomas[9,10]. The fusion regions of translocation products are specifically expressed by its corresponding tumors, thereby serving as targets of great potential for tumor specific therapies, including immunotherapy[11,12]. We[13,14] found that SYT-SSX fusion gene-derived peptides can be recognized by circulating CD8+ T cells in patients with synovial sarcoma and elicit HLA-restricted, tumor specific cytotoxic responses by in vitro stimulations. In this study, we conducted a phase I pilot trial of vaccination of a SYT-SSX-derived junction peptide in elected synovial sarcoma patients.

## Methods

### Peptide

A 9-mer peptide (SYT-SSX B: GYDQIMPKK) spanning the SYT-SSX fusion region was synthesized under good manufacturing practice (GMP) conditions by Multiple Peptide Systems (San Diego, CA). The identity of the peptide was confirmed by mass spectral analysis, and was shown to have more than 98% purity when assessed by high pressure liquid chromatography analysis. The peptide was delivered us in the form of a freeze-dried, sterile white powder. It was dissolved in 1.0 ml of physiological saline (Otsuka Pharmaceutical Co., Ltd., Tokyo, Japan) and stored at -80°C until just before usage. The affinity of the peptide to HLA-A24 molecules and its antigenicity were determined in previous studies[13,14].

### Eligibility

The study protocol was approved by the Clinical Institutional Ethical Review Board of the Medical Institute of Bioregulation, Sapporo Medical University, Japan. Eligible patients were those (i) who have histologically and genetically confirmed, unresectable synovial sarcoma (SYT-SSX1 or SYT-SSX2 positive), (ii) HLA-A*2402 positive, (iii) between 20 and 70 years old, (iv) ECOG performance status between 0 and 3, and (v) who gave informed consent. Exclusion criteria included (i) prior chemotherapy, steroid therapy, or other immunotherapy within the past 4 weeks, (ii) presence of other cancers that might influence the prognosis, (iii) immunodeficiency or a history of splenectomy, (iv) severe cardiac insufficiency, acute infection, or hematopoietic failure, (v) ongoing breast-feeding, (vi) unsuitability for the trial based on the clinical judgment of the doctors involved. This study was carried out at the Department of Orthopaedic Surgery, Sapporo Medical University Hospital from June 2003 until the end of September 2004.

### Vaccination schedule

Vaccinations with SYT-SSX B peptide were administered subcutaneously into the upper arm six times at 14-day intervals. In order to set up a dose-escalation trial, the patients were separated into the two groups. Each group included three patients. Those from group 1 received 0.1 mg and group 2 participants received 1.0 mg.

### Delayed-type hypersensitivity (DTH) skin test

Delayed-type hypersensitivity (DTH) skin test was performed at each vaccination. The peptide (10 μg) solution in physiological saline (0.1 ml) or physiological saline alone (0.1 ml) were separately injected intradermally into the forearm. A positive reaction was defined as a diameter of erythema of more than 4 mm, 48 hr after the injection.

### Toxicity evaluation

Patients were examined closely for signs of toxicity during and after vaccination. Adverse events were recorded using the National Cancer Institute Common Toxicity Criteria (NCI-CTC).

### Clinical response evaluation

Physical examinations and hematological examinations were monitored before and after each vaccination. Tumor size was evaluated by computed tomography (CT) scans before treatment, and again after three vaccinations, and then at the end of the study period. A complete response (CR) was defined as complete disappearance of all measurable diseases. A partial response (PR) was defined as a >= 50% decrease from the baseline in size of all measurable lesions (sum of products of maximal perpendicular diameters) lasting for a period of at least 4 weeks. Progressive disease (PD) was defined as an increase in the sum of the bi-dimensional measurements of all known disease sites by at least 25% or by the appearance of new lesions. No change (NC) was defined as the absence of matched criteria for CR, PR, or PD.

### Tetramer staining

HLA-A24/peptide tetramers (HLA-A24/B, HLA-A24/R49.2, and HLA-A24/HIV) were previously constructed [13-15]. Flowcytometric analysis was performed by taking peripheral blood mononuclear cells (PBMCs) from patients. PBMCs were taken at pre-vaccination and again one week after 1^st^, 3^rd^, and 6^th ^vaccination. Cells were stained with PE-labeled tetramers at 37°C for 20 min and a FITC-conjugated anti-CD8 mAb (Becton Dickinson) at 4°C for 30 min. Analysis of stained PBMCs was performed using FACScan (Becton Dickinson) and CellQuest software (Becton Dickinson). The frequency of CTL precursors was calculated as the number of tetramer positive cells / the number of CD8^+ ^cells.

### CTL induction

Cytotoxic T lymphocytes (CTLs) were induced from the PBMCs of patients using SYT-SSX B peptides according to the method described before[13,14]. The cytotoxic activity was evaluated by 6-h ^51^Cr release assay[13]. As target cells, synovial sarcoma cell lines (Fuji, HS-SY-II, and SW982), an erythroleukemia cell line (K562), and a T-B Lymphoblast hybrid transfected with HLA-A*2402 (T2-A*2402) were used. Fuji and HS-SY-II were both HLA-A24 and SYT-SSX positive lines. SW982 and K562 were both HLA-A24 and SYT-SSX negative lines used as controls. T2-A*2402 cells were used to determine peptide-specific cytotoxicity by pulsation with SYT-SSX B or HIV peptide before labeling. The stimulated CD8^+ ^T cells were mixed with the labeled target cells. After a 6-h incubation period at 37°C, the release of the ^51^Cr label was measured by collecting the supernatant, followed by quantification in an automated gamma counter. The percentage of specific cytotoxicity was calculated as the percentage of specific ^51^Cr release: [(experimental ^51^Cr release - spontaneous ^51^Cr release) / (maximum ^51^Cr release - spontaneous ^51^Cr release)] × 100. Maximum ^51^Cr release was measured by incubating the labelled target cells with 2% NP-40, instead of the stimulated CD8^+ ^T cells. CTL induction was determined as successful when specific cyotoxicity of 10% or more was achieved on Fuji, HS-SY-II, and SYT-SSX B peptide-pulsed T2-A*2402 cells.

## Results

### Patient profiles

Six patients were enrolled in the study (Table 1). There were four men and two women with an average age of 34.7 years old (range 21–69 years). All patients had multiple metastatic lesions of the lung. A six-time vaccination schedule was completed in three patients, while the remaining three discontinued the vaccination regimen because of rapid disease progression. None of the treatment interruptions were due to the adverse effects of the vaccination.

**Table 1:** Profiles of participants and clinical resoponses

Patient no.	Age	Gender	Dose of peptide (mg)	Number of vaccination	Adverse events	DTH skin test	Evaluation of CT images
1	69	M	0.1	1	-	-	PD
2	32	M	0.1	3	-	-	PD
3	21	F	0.1	6	-	-	PD
4	21	M	1.0	6	-	-	PD
5	39	F	1.0	6	Fever	-	NC
6	26	M	1.0	4	-	-	PD

PD: progressive disease, NC: no change

### Safety and DTH skin test

One patient (case 5) experienced slight fever (grade 1) after the first vaccination. No other adverse events were observed during vaccination. DTH skin test was performed at each vaccination and assessed 48 hr later. Reactions were determined as negative in all patients.

### Clinical response

**Figure 1:**
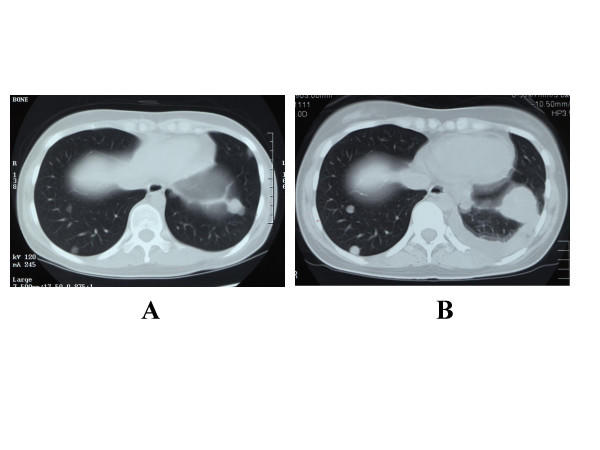
CT scan image of the lung of case 3 patient. A: Before vaccination (May 15, 2003). B: After the third vaccination (June 18, 2003). Rapid growth of the metastatic tumors and pleural effusion were seen.

**Figure 2:**
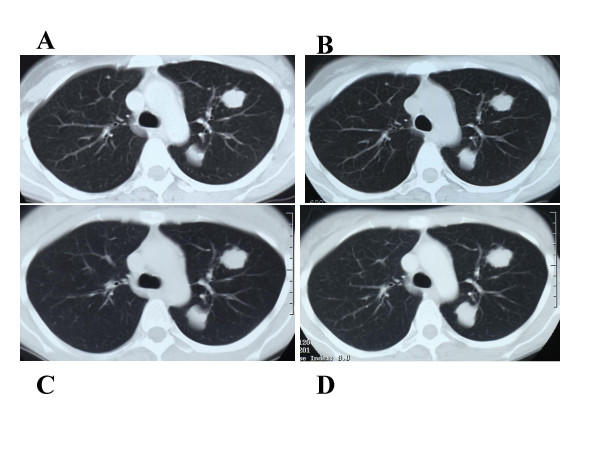
CT scan image of the lung of case 5 patient. A: Before vaccination (July 8, 2003). B: After the first vaccination (July 22, 2003). C: After the third vaccination (August 19, 2003). D: After the sixth vaccination (September 16, 2003). The metastatic tumors appeared to be dormant after the first vaccination.

Recognized disease progression occurred in five out of six patients during the vaccination period (Table 1, Fig. 1). In contrast, one patient (case 5) showed no such rapid progression (Table 1, Fig. 2). These patients, except in case 1, had received systemic multidrug chemotherapy from one to four months before enrolling on this study.

### Tetramer analysis and CTL induction

**Figure 3:**
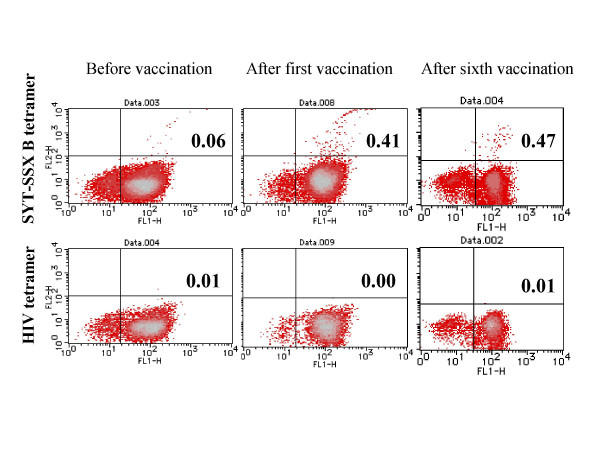
Frequency of CTLs analyzed by HLA-A24/peptide tetramers in the case 4 patient. Frequencies of each analysis were described in Table 2.

Peptide-specific immunological responses were evaluated in five patients by using HLA-A24/peptide tetramer analysis and in vitro CTL induction. As determined by flowcytometric analysis using HLA/peptide tetramers (Table 2), frequencies of CTLs specific for SYT-SSX B peptide were shown to be at background levels (less than 0.1%) in three patients prior to vaccination. Those frequencies increased after the first (cases 4 and 6) and the third vaccination (case 2) (Fig. 3). In the remaining two patients (cases 3 and 5), SYT-SSX B peptide-specific CTLs existed beyond the background levels before vaccination. Of these, B peptide-specific CTL frequencies increased slightly in case 2 upon a series of vaccinations. On the contrary, the CTL frequencies in peripheral blood decreased to the background level after the third vaccination in case 5, whose metastatic diseases remained stable during the vaccination period. For comparison, tetramers with irrelevant peptides were constructed as internal controls and utilized in four patients. Notably, CTL frequencies reacting to those irrelevant tetramers remained under the background level during the course of vaccinations in all patients.

**Table 2:** HLA-A24/peptide tetramer analysis

case	Pre-vaccination	After 1st vac.	After 3rd vac.	After 6th vac.
2	0.02/N.D	0.02/N.D	**3.05**/N.D	N.D
3	**0.42**/0.02*	**0.49**/0.02	**0.52**/0.02	**0.62**/0.01
4	0.06/0.01	**0.41**/0.00	**0.36**/0.01	**0.47**/0.01
5	**0.50**/0.06	**0.52**/0.01	0.09/0.00	0.03/0.02
6	0.02/0.01	**0.15**/0.01	0.08/0.00	N.D

N.D: not determined, *B peptide tetramer / Control peptide tetramer

HLA-A24/R49.2 pepitde tetramer was used as control in the case 3 patient.

HLA-A24/HIV pepitde tetramer was used as control in the other patients.

**Figure 4:**
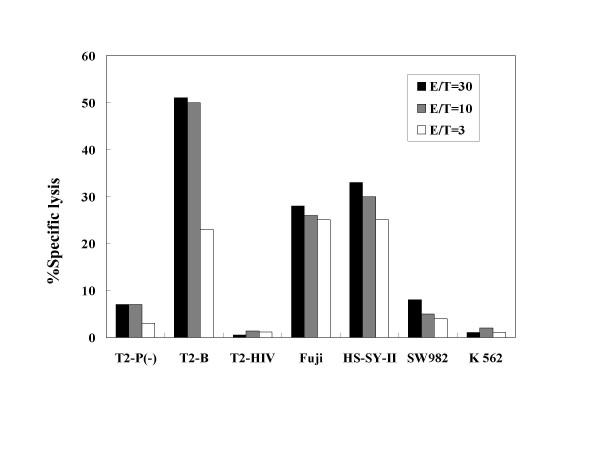
Cytotoxicity of CTLs induced from the case 4 patient. T2-P(-): T2-A*2402 cells without peptide pulsation, T2-B: T2-A*2402 cells pulsed with SYT-SSX B peptide, T2-HIV: T2-A*2402 cells pulsed with HIV peptide. Specific cytotoxicity was observed against T2-A*2402 cells pulsed with SYT-SSX B peptide, and synovial sarcoma cell lines expressing HLA-A24 and SYT-SSX (Fuji and HS-SY-II). E/T: Effecter cells/target cells ratio.

Table 3 depicts the results of CTL induction by in vitro stimulations with SYT-SSX B peptide. Before vaccination, CTLs specific for SYT-SSX B peptide were successfully induced from one patient (case 2) who showed a high frequency of CTL precursors. After the first or third vaccination, CTLs were induced from four of five patients. Figure 4 represents the results of cytotoxicity assay. As shown, CTLs induced from the case 4 patient exhibited cytotoxic activities against T2-A*2402 cells pulsed with SYT-SSX B peptide, and synovial sarcoma cell lines expressing HLA-A24 and SYT-SSX (Fuji and HS-SY-II) in various effecter/target ratios examined. In contrast, the cytotoxity was less than 10% against T2-A*2402 cells without peptide pulsation, those pulsed with irrelevant HIV peptide, and tumor cells lacking HLA-A24 and SYT-SSX (SW982 and K562). These findings suggest that induction of peptide-specific immune responses in patients with synovial sarcoma, who received the SYT-SSX junction peptide vaccine.

**Table 3:** Induction of peptide specific CTLs

case	Pre-vaccination	After 1st vac.	After 3rd vac.	After 6th vac.
2	Failure	Failure	**Success**	N.D
3	**Success**	**Success**	**Success**	Failure
4	Failure	**Success**	**Success**	N.D
5	Failure	**Success**	Failure	Failure
6	Failure	Failure	Failure	N.D

N.D: not determined

## Discussion

The present study was designed to evaluate the in vivo immunological property of a 9-mer SYT-SSX junction peptide in patients with disseminated synovial sarcoma. A total of 16 vaccinations of the peptide in six patients revealed (i) no serious adverse effects in any case, (ii) suppression of tumor progression in one patient, (iii) increases in the frequency of peptide-specific CTLs in three patients and a decrease in one patient, and (iv) successful induction of peptide-specific CTLs from four patients. These findings suggest that the SYT-SSX junction peptide is safe to use as a vaccine and also has the property to evoke in vivo immunological responses.

With respect to the clinical efficacy, none of the enrolled patients showed signs of tumor remission. However, in the case 5 patient, tumors showed some dormancy during the vaccination period, in comparison to the other five patients in whom tumors grew rapidly from the early phase of the regimen. The case 5 patient had received one cycle of systemic chemotherapy four months before enrollment, making the possibility of residual chemotherapeutic efficacy unlikely. The other five patients, except in case 1, had received systemic chemotherapy one to two months before perticipation. Notably, frequency of circulating peptide-specific CTLs decreased in the case 5 patient, while CTL frequencies remained unchanged or increased significantly in the other four patients examined. Decrease in circulating CTLs in the case 5 patient may have resulted from accumulation of CTLs at the tumor sites, although biopsy of the tumors was not performed.

Vaccination trials of fusion gene-derived peptides have been reported with BCR-ABL in 12 patients with chronic myelogenous leukemia[16], EWS-FLI1 in 12 patients with Ewing's sarcoma[17], and PAX3-FKHR in four patients with alveolar rhabdomyosarcoma[17]. In addition, Matsuzaki et al.[18] reported a case of a synovial sarcoma patient who were treated with autologous dendritic cells pulsed with a mixture of SYT-SSX junction peptides (8–16 mer). In these studies, tumor remission was noted only in one patient with Ewing's sarcoma where IL-2 was concomitantly administered. These findings together with the results of our present trial have indicated the limited therapeutic efficacy of natural junction peptides. In this regard, we discovered improved in vitro immunogenicity of the SYT-SSX junction peptide by the substitution of an HLA-A24 anchor residue (position 9)[14]. Clinical study of this anchor-substituted peptide is currently underway. Besides modification of the peptide itself, concurrent use of adjuvants and cytokines, and adoptive T cell or/and dendritic transfer should further improve the therapeutic efficacy[8,19]. Also, it is important to determine the appropriate timing of vaccination and proper endpoints of clinical studies.

To monitor the immunological responses, we used HLA/peptide tetramer and in vitro CTL induction. Other monitoring procedures such as ELISPOT assay should provide further information. Nevertheless, the use of an internal control tetramer with HIV peptide added strength in this present comparative analysis. As shown in Table 2 and Fig. 4, reactivity of circulating T cells to HIV peptide tetramer remained under the background level throughout the vaccination period.

Due to the rarity and highly malignant nature of synovial sarcoma, three out of six patients failed to complete the six-time vaccination regimen. Such difficulty in continuation of vaccination has also been found in a trial of patients with Ewing's sarcoma and alveolar rhabdomyosarcoma[17]. Another limitation in the current study is lack of analysis on immunological significance of SYT-SSX variants (SYT-SSX1 and SYT-SSX2). This question should be addressed in a larger scale analysis.

## Conclusions

This is the first clinical trial of SYT-SSX fusion gene-derived peptide in patients with synovial sarcoma. The present trial demonstrated the safety and immunogenic property of the peptide. Modification of both the peptide itself and the related protocol is required to further improve the therapeutic efficacy.

## Abbreviations

CTL; cytotoxic T lymphocyte, HLA; human leukocyte antigen, MHC; major histocompatibility complex, PBMC; peripheral blood mononuclear cell

## Competing interests

The author(s) declare that they have no competing interests.

## Authors' contributions

SK1 and YS carried out vaccinations and clinical evaluations. KI, TT, SK1,2, HA and KS carried out monitoring procedures. TW, SN, HS, HI, TT, HH, TI, ST, NS, and TY made substantial contributions to the design of the study.

## References

[B1] Fisher C, de Bruijn DR, Geurts van Kessel A, Fletcher CDM, Unni KK, Mertens F (2002). Synovial sarcoma. World Health Organization classification of tumours Pathology and genetics of tumours of soft tissue and bone.

[B2] Deshmukh R, Mankin HJ, Singer S (2004). Synovial sarcoma: the importance of size and location for survival. Clin Orthop.

[B3] Guillou L, Benhattar J, Bonichon F, Gallagher G, Terrier P, Stauffer E, Somerhausen Nde S, Michels JJ, Jundt G, Vince DR, Taylor S, Genevay M, Collin F, Trassard M, Coindre JM (2004). Histologic grade, but not SYT-SSX fusion type, is an important prognostic factor in patients with synovial sarcoma: a multicenter, retrospective analysis. J Clin Oncol.

[B4] Ladanyi M, Antonescu CR, Leung DH, Woodruff JM, Kawai A, Healey JH, Brennan MF, Bridge JA, Neff JR, Barr FG, Goldsmith JD, Brooks JS, Goldblum JR, Ali SZ, Shipley J, Cooper CS, Fisher C, Skytting B, Larsson O (2002). Impact of SYT-SSX fusion type on the clinical behavior of synovial sarcoma: a multi-institutional retrospective study of 243 patients. Cancer Res.

[B5] Okcu MF, Munsell M, Treuner J, Mattke A, Pappo A, Cain A, Ferrari A, Casanova M, Ozkan A, Raney B (2003). Synovial sarcoma of childhood and adolescence: a multicenter, multivariate analysis of outcome. J Clin Oncol.

[B6] Mocellin S, Mandruzzato S, Bronte V, Lise M, Nitti D (2004). Part I: Vaccines for solid tumours. Lancet Oncol.

[B7] Ribas A, Butterfield LH, Glaspy JA, Economou JS (2003). Current developments in cancer vaccines and cellular immunotherapy. J Clin Oncol.

[B8] Ayyoub M, Taub RN, Keohan ML, Hesdorffer M, Metthez G, Memeo L, Mansukhani M, Hibshoosh H, Hesdorffer CS, Valmori D (2004). The frequent expression of cancer/testis antigens provides opportunities for immunotherapeutic targeting of sarcoma. Cancer Immun.

[B9] Bennicelli JL, Barr FG (2002). Chromosomal translocations and sarcomas. Curr Opin Oncol.

[B10] Rabbitts TH, Stocks MR (2003). Chromosomal translocation products engender new intracellular therapeutic technologies. Nat Med.

[B11] Maki RG (2001). Soft tissue sarcoma as a model disease to examine cancer immunotherapy. Curr Opin Oncol.

[B12] Le Poole IC, Gerberi T, Kast WM (2002). Emerging strategies in tumor vaccines. Curr Opin Oncol.

[B13] Sato Y, Nabeta Y, Tsukahara T, Hirohashi Y, Syunsui R, Maeda A, Sahara H, Ikeda H, Torigoe T, Ichimiya S, Wada T, Yamashita T, Hiraga H, Kawai A, Ishii T, Araki N, Myoui A, Matsumoto S, Umeda T, Ishii S, Kawaguchi S, Sato N (2002). Detection and induction of CTLs specific for SYT-SSX-derived peptides in HLA-A24(+) patients with synovial sarcoma. J Immunol.

[B14] Ida K, Kawaguchi S, Sato Y, Tsukahara T, Nabeta Y, Sahara H, Ikeda H, Torigoe T, Ichimiya S, Kamiguchi K, Wada T, Nagoya S, Hiraga H, Kawai A, Ishii T, Araki N, Myoui A, Matsumoto S, Ozaki T, Yoshikawa H, Yamashita T, Sato N (2004). Crisscross CTL induction by SYT-SSX junction peptide and its HLA-A*2402 anchor substitute. J Immunol.

[B15] Sato Y, Sahara H, Tsukahara T, Kondo M, Hirohashi Y, Nabeta Y, Kawaguchi S, Ikeda H, Torigoe T, Ichimiya S, Tamura Y, Wada T, Yamashita T, Goto M, Takasu H, Sato N (2002). Improved generation of HLA class I/peptide tetramers. J Immunol Methods.

[B16] Pinilla-Ibarz J, Cathcart K, Korontsvit T, Soignet S, Bocchia M, Caggiano J, Lai L, Jimenez J, Kolitz J, Scheinberg DA (2000). Vaccination of patients with chronic myelogenous leukemia with bcr-abl oncogene breakpoint fusion peptides generates specific immune responses. Blood.

[B17] Dagher R, Long LM, Read EJ, Leitman SF, Carter CS, Tsokos M, Goletz TJ, Avila N, Berzofsky JA, Helman LJ, Mackall CL (2002). Pilot trial of tumor-specific peptide vaccination and continuous infusion interleukin-2 in patients with recurrent Ewing sarcoma and alveolar rhabdomyosarcoma: an inter-institute NIH study. Med Pediatr Oncol.

[B18] Matsuzaki A, Suminoe A, Hattori H, Hoshina T, Hara T (2002). Immunotherapy with autologous dendritic cells and tumor-specific synthetic peptides for synovial sarcoma. J Pediatr Hematol Oncol.

[B19] Rosenberg SA, Yang JC, Restifo NP (2004). Cancer immunotherapy: moving beyond current vaccines. Nat Med.

